# Saudi experts statement on advancing multiple myeloma treatment: the evolving role of bispecific antibodies

**DOI:** 10.3389/fmed.2025.1730261

**Published:** 2026-01-21

**Authors:** Ghazi S. Alotaibi, Ahmad Alsaeed, Majed Alahmadi, Abdullah Alamer, Abdullah S. Al Saleh, Ahmed Salleh Barefah, Saud Alhayli, Enas Yahya Mutahar, Omar Abduljalil, Hesham Kiwan, Ayman Alhejazi

**Affiliations:** 1 Department of Medicine (Oncology Center) College of Medicine, King Saud University, Riyadh, Saudi Arabia; 2 Adult Hematology and HSCT, Princess Noorah Oncology Centre, King Abdulaziz Medical City, Ministry of National Guard Health Affairs-Western Region, Jeddah, Saudi Arabia; 3 College of Medicine, King Saud Bin Abdulaziz University for Health Sciences (KSAU-HS), Ministry of National Guard Health Affairs-Western Region, Jeddah, Saudi Arabia; 4 King Abdullah International Medical Research Centre (KAIMRC), Ministry of National Guard Health Affairs-Western Region, Jeddah, Saudi Arabia; 5 King Faisal Specialist Hospital and Research Center, Riyadh, Saudi Arabia; 6 College of Medicine, King Saud bin Abdulaziz University for Health Sciences, Riyadh, Saudi Arabia; 7 King Abdullah International Medical Research Center, Riyadh, Saudi Arabia; 8 Division of Hematology & HSCT, Department of Oncology, King Abdulaziz Medical City, Ministry of National Guard-Health Affairs, Riyadh, Saudi Arabia; 9 Hematology Department, Faculty of Medicine, King Abdulaziz University, Jeddah, Saudi Arabia; 10 Hematology Research Unit, King Fahd Medical Research Center, King Abdulaziz University, Jeddah, Saudi Arabia; 11 Adult Hematology and HSCT, King Fahad Specialist Hospital, Dammam, Saudi Arabia; 12 Pfizer, Saudi Arabia

**Keywords:** bispecific antibodies, CAR T-cell, myeloma, relapse/refractory, Saudi Arabia

## Abstract

**Introduction:**

The therapeutic landscape in multiple myeloma (MM) is rapidly evolving; however, a significant unmet need remains for patients with relapsed or refractory (RR) MM in Saudi Arabia. Although chimeric antigen receptor T-cell (CAR-T) therapies for myeloma have received approval in the region, their accessibility remains limited, and many patients are ineligible due to factors such as advanced age, comorbidities, rapidly progressive disease, or logistical barriers. Bispecific antibodies (BsAbs) are emerging as a promising option in Saudi Arabia, but the paucity of region-specific clinical evidence underscores the critical need for tailored, evidence-based guidance to optimize their application in clinical practice.

**Methods:**

A group of experts in MM based in Saudi Arabia convened to align on optimal use of BsAbs in clinical practice based on their experience and evidence from a literature analysis. A modified Delphi methodology with prespecified acceptance threshold of ≥70% was used to generate expert recommendations.

**Results:**

Agreement was achieved in 30 of 35 statements (86%) across four broad areas: patient selection, sequencing and dosing, monitoring, and efficacy outcomes. Experts agreed that BsAbs should be positioned in triple-class refractory MM. Positive clinical trial outcomes observed with BsAbs, including high overall response rates, prolonged progression-free survival and overall survival underscore the importance of selecting BsAbs with proven efficacy. While CAR T-cell therapy is only recently approved for MM in Saudi Arabia, BsAbs are preferred for patients with rapidly progressing disease due to CAR T-cell product manufacturing delays.

**Conclusion:**

BsAbs are an effective treatment option for those with triple-class refractory MM and this manuscript provides a framework to support their optimal use in Saudi Arabia.

## Introduction

The therapeutic landscape in multiple myeloma (MM) is rapidly evolving (1). Immunomodulatory drugs, proteasome inhibitors and anti-CD38 monoclonal antibodies (mAbs) have all emerged as valuable therapeutic options (2, 3). Combining agents with different targets and anti-myeloma activity has improved survival rates (2–5).

Despite therapeutic advancements, leading to prolonged remission times, high-risk patients with MM may experience limited benefit from current treatment modalities (6, 7). Furthermore, relapse is common with diminishing periods of remission due to drug resistance and the development of refractory disease, with treatment-related toxicities, comorbidities and frailty leading to poor outcomes (7). Patients refractory to an immunomodulatory drug, proteasome inhibitor and anti-CD38 mAb (triple-class refractory) have a median overall survival (OS) of <1 year (8–10).

The emergence of chimeric antigen receptor (CAR) T-cells and bispecific antibodies (BsAbs) has presented options for patients with relapsed/refractory MM (RRMM), including those who are triple-class refractory, as well as potential application in earlier lines of treatment (11–14). Both CAR T-cell therapies and BsAbs aim to redirect T-cell activity toward specific tumor antigens, yet they differ in their biological mechanisms, resistance profiles, and regulatory approval status (15, 16). As a result, careful patient selection, optimal timing, and appropriate sequencing of these therapies are critical to maximizing their clinical benefit (15, 17).

The use of BsAbs in contemporary practice has emerged as a particularly important clinical issue given the recent approval of these agents and their increasing use globally (18). Four BsAbs (teclistamab, elranatamab, talquetamab and linvoseltamab) have been recently approved by the United States Food and Drug Administration (FDA) for use in patients with RRMM who have received at least four previous lines of therapy, including immunomodulatory agent, proteasome inhibitor and an anti-CD38 mAb (16). BsAbs are engineered to bind to T-cells and a specific tumor cell target antigen, leading to immune cell–mediated death of tumor cells (19). Clinical trial data support the single-agent activity of BsAbs targeting the B-cell maturation antigen (BCMA), G protein-coupled receptor, class C, group 5, member D (GPRC5D), and Fc receptor-homolog 5 (FcRL5) antigens (20–25). Newer BsAbs are also under active investigation and have the potential to further impact the treatment paradigm of RRMM in the near future (26, 27).

It is crucial that BsAbs are used effectively in the right patients and at the right time, which requires careful consideration of patient-specific and tumor-specific factors, anticipated toxicities, and the sequencing of available therapies (16, 17). International guidelines provide recommendations for the use of BsAbs but the availability of these agents, along with other MM therapies (e.g., CAR T-cells), varies across regions (13, 28). Patients in Saudi Arabia have access to two commercially available anti-BCMA and one anti-GPRC5D monoclonal antibody, and two centers provide anti-BCMA CAR-T therapy. Given the growing use of BsAbs in MM and the absence of Saudi-specific guidelines, this paper reviews international evidence and provides expert recommendations tailored to regional resources and patient needs.

## Methods

An initial meeting took place on 28 May 2025 to define the purpose of the document and the methodological approach. The three aims of the manuscript were: to evaluate current practices internationally, to evaluate current clinical practice in Saudi Arabia, and to consider future directions and practice for the use of BsAbs in patients with MM.

A comprehensive literature search was completed using online databases (MEDLINE, CENTRAL and EMBASE) to identify relevant literature on the use of BsAbs in MM, including international publications. The search strategy focused on English language articles, human studies, articles in adult populations (aged 18 years and over) and articles published between 2020 and 2025. Meta-analyses, randomized controlled trials (RCTs) and single-arm prospective studies were eligible for inclusion; guidelines and literature reviews were also included to ensure a clear practice focus. The search query specifically targeted BsAbs used in the treatment of multiple myeloma, including agents such as teclistamab, elranatamab, linvoseltamab, ABBV-383, F182112, talquetamab, forimtamig, and cevostamab.

The literature review was used as a basis to inform the development of expert recommendations for the use of BsAbs in patients with MM. No pilot phase was undertaken because the consensus process was structured to begin with a comprehensive literature review and an initial scoping meeting, which provided sufficient foundation for expert recommendations. The expert panel comprised 10 physicians members of the Saudi Myeloma Working Group who are selected for their clinical experience, academic work, and national myeloma involvement. Four topics were selected to focus the modified Delphi approach: patient selection, sequencing and dosing, monitoring, and efficacy outcomes. A modified Delphi approach (29) was employed to achieve agreement on the 35 statements devised by the expert panel. Agreement percentages were achieved using a virtual survey (see Supplementary Table 1) delivered to all members of the expert panel. Panel members graded the level of agreement with each of the 35 statements as strongly agree, agree, neutral, disagree or strongly disagree. Based on the findings of the initial online survey, statement results were discussed in a virtual meeting held on 29 June 2025 with all members of the expert panel, with a second round of grading of the strength of the revised statements (see Boxes 1–4). Expert recommendations were categorized as strong or weak depending on the criteria noted in Table 1. Where agreement of at least ≥70% was not achieved, the core group discussed and aligned on the strength of recommendation linked to specific statements.

Box 1 **Expert recommendations for patient selection for BsAbs in multiple myeloma.**

**1.1 Effective treatment options for triple-class RRMM represent a significant clinical unmet need**.

*STRONG recommendation; 90% agreement*

**1.2 Criteria for selecting patients for BsAbs should include fitness (ECOG performance status), prior therapies, and biomarkers (ferritin level, CRP and IL-6), with special considerations for high-risk cytogenetics and frail patients**.

*STRONG recommendation; 100% agreement*

**1.3 The suitability of high-risk patients, such as those with high-risk cytogenetics, early relapse, extramedullary disease, etc., for bispecific antibody therapy should be discussed early, prior to relapse, with the medical team so an agreed pathway can be established**.

*STRONG recommendation; 90% agreement*

**1.4 Existing frailty assessment tools (such as ECOG performance status) should be incorporated when developing treatment plans**.

*STRONG recommendation; 100% agreement*

**1.5 Although CAR T-cell therapy has been approved in Saudi Arabia, it is not yet readily available. When accessible, patients with rapidly progressing disease may be more appropriate for bispecific antibody therapy, given the extended manufacturing time required for anti-BCMA CAR T-cell products**.

*STRONG recommendation; 90% agreement*

**1.6 BsAbs should be positioned in triple-class refractory MM. Additional experience is required to inform evidence-based decisions on treatment sequencing for triple-class RRMM**.

*STRONG recommendation; 100% agreement*

Box 2 **Expert recommendations for sequencing and dosing of BsAbs in multiple myeloma.**

**2.1 Optimal dosing and schedule adjustments should be based on patient response and tolerance**.

*STRONG recommendation; 90% agreement*

**2.2 Bridging therapy before CAR T-cell therapy should be standardized**

*WEAK recommendation; 60% agreement*

**2.3 BsAbs are a preferred option for bridging therapy before CAR T-cell therapy compared to other available methods**

*WEAK recommendation; 60% agreement*

**2.4 Switching the target antigen between bridging therapy and CAR T-cell therapy (e.g., using a GPRC5D-targeting bispecific antibody before BCMA-directed CAR T-cell therapy) may be considered**.

*STRONG recommendation; 90% agreement*

**2.5 For patients with RRMM who have not received CAR T-cell therapy, the optimal treatment option is a BCMA-targeting BsAb**

*STRONG recommendation; 70% agreement*

**2.6 For patients with RRMM who have previously received BCMA-directed CAR T-cell therapy and subsequently relapsed, a BCMA-targeting BsAb represents an optimal treatment option**

WEAK *recommendation; 30% agreement*

**2.7 For patients with RRMM who received BCMA-directed CAR T-cell therapy and were bridged with a BsAb, switching to a bispecific that targets a different antigen remains a strong treatment option after relapse**.

*STRONG recommendation; 90% agreement*

**2.8 In-patient hospitalization is recommended during the initiation phase of BsAb therapy for patients at high risk of cytokine release syndrome (CRS) or neurotoxicity, to ensure prompt recognition and management of adverse events, with outpatient initiation considered only in centers equipped with appropriate monitoring and emergency response capabilities**.

*STRONG recommendation; 100% agreement*

**2.9 The up-titration schedule of BsAbs should follow a gradual escalation approach, particularly during the initial dosing phase, to minimize the risk of immune-related toxicities such as CRS and ICANS**.

*STRONG recommendation; 90% agreement*

**2.10 The transition to maintenance dosing should be based on the patient's response during the up-titration phase, including resolution of any immune-related adverse events (e.g., CRS or ICANS), with careful consideration of any ongoing toxicities**.

*STRONG recommendation; 100% agreement*

**2.11 For most BsAbs, the maintenance phase typically involves a reduced dosing frequency following the initial up-titration period, with administration every 2–4 weeks**.

*STRONG recommendation; 80% agreement*

**2.12 Patient adherence and quality of life should be prioritized during the maintenance phase, and the dosing schedule should aim to reduce treatment burden without compromising efficacy**.

*STRONG recommendation; 100% agreement*

Box 3 **Expert recommendations for monitoring BsAbs in multiple myeloma.**

**3.1 The incidence and severity of CRS, ICANS, and infections in clinical practice are significant, with long-term safety concerns including cytopenia and immune-related effects**.

*STRONG recommendation; 70% agreement*

**3.2 Centers administering bispecific antibodies should establish protocols for monitoring cytokine levels, ferritin, and CRP, to detect early signs of CRS and facilitate timely intervention**.

*STRONG recommendation; 90% agreement*

**3.3 CRS is likely to occur soon after initiation (within 1 week) and CRS with late doses is very uncommon**.

*STRONG recommendation; 100% agreement*

**3.4 Patients should be aware of the risk of CRS and ICANS prior to initiation of bispecific agents and know who to contact if they experience symptoms**.

*STRONG recommendation; 100% agreement*

**3.5 Best practices for grading and management of CRS and ICANS should be established in each center, in line with international guidelines, such as those outlined by the American Society of Hematology (ASH) and the American Society for Transplantation and Cellular Therapy (ASTCT)**.

*STRONG recommendation; 100% agreement*

**3.6 During the up-titration phase, patients should be closely monitored for early signs of CRS and ICANS, with assessments performed at frequent intervals (every 4–6 h), particularly within the first 24–48 h following each dose escalation**.

*STRONG recommendation; 100% agreement*

**3.7 Long-term monitoring during the maintenance phase should include assessments every 1–3 months of disease response (e.g., via laboratory markers, imaging studies, and bone marrow aspiration and biopsies) and adverse events, such as hematologic toxicity or infections**.

*STRONG recommendation; 100% agreement*

**3.8 Hospital ward and on-call medical, ambulatory care, nursing, and pharmacy teams should be familiar with CRS and ICANS treatment and management (including staging and grading)**.

*STRONG recommendation; 100% agreement*

**3.9 Biomarkers, such as ferritin, CRP and IL-6, play a role in predicting CRS/ICANS severity**.

*STRONG recommendation; 100% agreement*

**3.10 Infections should not be a barrier to treatment. Adequate infection management is vital to avoid treatment interruption. Recurrent infections are common and should be managed prophylactically and cautiously. All patients should receive IVIG and be initiated on antiviral, anti-PCP and antifungal prophylaxis**.

*STRONG recommendation; 100% agreement*

**3.11 Bispecific antibody treatment should be started in tertiary centers, and after 1–2 cycles the patient can be seen in a secondary hospital**

*STRONG recommendation; 80% agreement*

**3.12 G-CSF is recommended for consideration in patients with Grade 3–4 neutropenia**.

*STRONG recommendation; 100% agreement*

Box 4 **Expert recommendations on efficacy outcomes of BsAbs in multiple myeloma.**

**4.1 Given the significant impact of efficacy on treatment outcomes, it is crucial to consider the efficacy profiles of bispecific antibodies when making treatment decisions**.

*STRONG recommendation; 100% agreement*

**4.2 The positive outcomes observed in clinical trials, including high overall response rates, prolonged progression-free survival and overall survival underscore the importance of selecting BsAbs with proven efficacy**.

*STRONG recommendation; 100% agreement*

**4.3 The predictive value that MRD negativity, evidenced in clinical trials, highlights its importance when selecting BsAbs for treatment decision-making in MM**.

*STRONG recommendation; 90% agreement*

**4.4 Differences in the patient populations enrolled and efficacy results between various BsAbs have been noted in international randomized controlled trials and real-world evidence that may inform treatment choices**.

*STRONG recommendation; 90% agreement*

**4.5 Real-world studies evaluating BsAbs in RRMM need to be prioritized in the patient population in Saudi Arabia**.

*STRONG recommendation; 100% agreement*

**Table 1:** Criteria for classification of the strength of expert recommendations.

**Strong recommendation**	**Weak recommendation**
Data derived from randomized controlled trials or meta-analysis	Data derived from sources with a lower level of evidence
Agreement among ≥70% of respondents (proportion of strongly agree + agree responses)	Agreement among 30–69% of respondents (proportion of strongly agree + agree responses)

The remainder of this document presents the findings of the expert panel, supported by relevant literature and regional insights for Saudi Arabia.

## Results

### Patient selection for BsAbs in multiple myeloma

The appropriate selection of patients for BsAb therapies is an important step in ensuring that their use can be optimized. Currently approved BsAbs are indicated for patients who have been exposed to four different lines of therapy (immunomodulatory agent, proteasome inhibitor and an anti-CD38 mAb), suggesting an important use for BsAbs in the patients with RRMM and those who are triple-class refractory. The low OS for this patient group compared with other patients, highlights the unmet clinical need for effective treatment options (8–10). The expert panel agreed that there is a pronounced unmet need for effective treatment in this patient group in Saudi Arabia (Box 1). Hence, the triple-class refractory patient group may be considered eligible for BsAb therapy, where efficacy has been demonstrated in pivotal trials (20–25).

The potential for maximizing the efficacy of BsAbs has been considered in relation to moving these therapies to earlier lines of treatment or through the use of combination therapy (16). However, other factors need to be considered when selecting patients to maximize the benefits of these therapies. The efficacy of BsAbs may be lower in high-risk patients, including those with high-risk cytogenetic features, early relapse and extramedullary disease and may also have an increased risk of toxicities related to therapy (30, 31). Frail patients and those with recurrent infections may need to be considered carefully for BsAb therapy due to the toxicity profiles of these agents, including the risk of cytokine release syndrome (CRS), immune effector cell-associated neurotoxicity (ICANS), prolonged cytopenia and infection risk (32). Different toxicity profiles of available BsAbs, as well as alternative treatment options (e.g., CAR T-cells) need to be considered in the context of the patient status and medical history to reduce risk and maximize benefit of treatment. The expert panel agreed that patient selection should include a range of criteria, including rapid disease progression, refractory status, fitness, prior therapies, biomarkers and considerations for high-risk features. The use of existing tools to evaluate performance status and frailty should be applied to guide decision-making, the panel emphasized that each case should be assessed individually, considering the unique clinical context and patient-specific factors.

With the recent approval of CAR T-cell therapy for multiple myeloma by the Saudi FDA, treatment selection between CAR T-cells and BsAbs warrants careful consideration. One challenge with CAR T-cell therapy remains the manufacturing and lead time, which can delay treatment initiation, particularly concerning in patients with rapidly progressing disease (33). In contrast, BsAbs offer an off-the-shelf option that enables prompt administration (34). Given these differences, the expert panel recommends prioritizing BsAbs in cases of aggressive disease, while considering CAR T-cell therapy in appropriately selected patients as regional access continues to expand.

### Sequencing and dosing of BsAbs in multiple myeloma

The sequencing of therapies in MM can have an important impact on outcomes. The expert panel agreed that both patient response to therapies and tolerance of doses used and prior treatment related toxicities should be factored into treatment sequencing and dosing decision-making (Box 2). The use of bridging therapy prior to CAR T-cell therapy is broadly recommended in published guidelines (13, 28) and the expert panel unanimously agreed that bridging therapy should be recommended.

The expert panel does not support standardized bridging therapy, as treatment choice must be individualized based on prior therapy exposure. Bridging therapy is recommended when feasible and should be tailored accordingly. BsAbs may be considered as bridging agents; however, the use of BCMA-targeting BsAbs prior to BCMA-directed CAR T-cell therapy is generally discouraged due to the risk of target antigen downregulation and T-cell exhaustion (35). In patients scheduled to receive BCMA-directed CAR T-cell therapy who require bridging therapy prior to infusion, a non-BCMA-targeting bispecific antibody—such as one directed against GPRC5D or FcRH5—may be preferred to reduce the risk of antigen escape and T-cell exhaustion (36, 37).

For patients with RRMM who have not received CAR T-cell therapy, it is essential that treatment decisions align with current international guidelines and local approval. For patients with RRMM ineligible for CAR T-cell therapy who have received at least four prior lines of therapy (including a proteasome inhibitor, an immunomodulatory agent, and an anti-CD38 mAb), BCMA-targeting BsAbs represent an FDA-approved therapeutic option (28). The expert panel agrees with this approach (1, 2).

During the initiation of BsAb therapy, the expert panel agrees that patients should be hospitalized until the first full dose is given. Patients can be discharged 24–48 h after the first full dose to allow for prompt recognition and management of adverse events, including CRS and neurotoxicity (38). The expert panel agreed that outpatient administration is only suitable where centers have appropriate monitoring and emergency response capabilities.

The expert panel endorses the use of non-BCMA-targeting BsAbs as the preferred option for patients with RRMM who relapse after BCMA-directed CAR T-cell therapy given evidence for objective, durable responses with these agents in this patient group (25, 36). Retreatment with BCMA-targeted therapies may still be appropriate in select cases, based on prior response, duration of remission, and continued BCMA expression (36).

The dosing of BsAbs should follow an up-titration approach with gradual escalation of the dose, as this is associated with a reduced risk of toxicities (39–41). Maintenance dose transition should be based on response during the up-titration phase, with resolution of immune-related adverse events (e.g., CRS or ICANS) (42). For most BsAbs, the maintenance phase comprises a reduction in dosing frequency compared with the up-titration period, with administration of the BsAb every 2–4 weeks, consistent with prescribing information and clinical trial protocols (13, 42). The expert panel noted that this is a rapidly evolving area and new products with different administrations will be approved for use in the coming months and years. Therefore, physicians must stay informed and responsive to updates as they emerge to ensure appropriate dosing strategies.

Patient adherence and quality of life should both be prioritized in the maintenance phase, with dosing schedules adjusted to ensure efficacy is maintained and to minimize treatment burden (43–46).

### Monitoring of BsAbs in multiple myeloma

Monitoring patients on BsAb therapy is crucial to reduce the risk of toxicities, initiate early management of adverse events, and to evaluate therapeutic efficacy. CRS is a common adverse event seen with both CAR T-cell therapy and BsAb therapy, comprising a systemic inflammatory reaction that may be life-threatening (47). CRS is seen in around three-quarters of patients receiving BsAbs, although the incidence is less common than with CAR T-cell therapy and grade 3 events are rare. Onset of CRS is typically during dose escalation or at the full first dose (within 1 week of initiation) (20, 22, 24). The expert panel agreed that centers should establish clear protocols for diagnosing and managing CRS, and monitoring cytokines, ferritin and C-reactive protein (CRP) levels to detect early signs of CRS (Box 3).

Neurotoxicity is another important adverse event associated with BsAb use in patients with RRMM and includes headaches, peripheral neuropathy and ICANS (13). ICANS is often associated with CRS and is thought to be due to passive diffusion of cytokines and T-cell trafficking in the central nervous system, couple with monocyte and macrophage activity (13). Manifestations of ICANS include seizures, intracranial hypertension and diffuse encephalopathy, with or without focal signs (47–49). Specific grading strategies have been developed for ICANS, including the Immune Effector Cell-Associated Encephalopathy (ICE) score, which is used by the American Society for Transplantation and Cellular Therapy (50). The expert panel agreed that there was a need for centers to adopt grading and management practices for CRS and ICANS consistent with international guidelines.

The expert panel agreed that in clinical practice, CRS, ICANS and infections have been reported with variable frequency and severity (47, 51). Long-term safety considerations may include persistent cytopenia and immune-mediated effects, though outcomes can differ based on patient and treatment factors (52). The incidence of both CRS and ICANS may be reduced through up-titration of dosing of BsAbs and this should be carefully implemented (49, 53, 54). This includes the need to close monitoring during up-titration of doses, with periodic assessments, support from multidisciplinary staff for CRS and ICANS detection and management, long-term monitoring and appropriate use of biomarkers (13, 49). Biomarkers such as ferritin, CRP and interleukin (IL)-6 can play a role in predicting the severity of CRS or ICANS and should be assessed accordingly, although a stronger evidence base would be valuable to support this recommendation (55). In addition, disease control before BsAb initiation can decrease the risk of CRS and ICANS and alternative options for patients at high risk are emerging such as prophylactic tocilizumab use (49, 51). The expert panel recommends that BsAb therapy should be initiated in tertiary care centers due to the risk of early adverse events such as CRS and ICANS. If treatment is well-tolerated over the first one to two cycles, subsequent treatment may be safely continued at a secondary hospital, provided appropriate monitoring capabilities are in place.

Although infection risk is an important consideration in patients with MM treated with BsAbs, the expert panel agreed that infections should not be a barrier to treatment. Adequate infection management is essential and can avoid the need to interrupt treatment, while prophylaxis is advocated to manage recurrent infections (13). As noted in recent guidance from the International Myeloma Working Group (36) the risk of infections with BsAbs targeted BCMA × CD3 can be significantly reduced by antimicrobial prophylaxis and intravenous immunoglobulin (IVIG) replacement therapy. The expert panel noted that patients should receive IVIG and be initiated on antiviral, anti-pneumocystis pneumonia (PCP) and antifungal prophylaxis to reduce infectious disease burden. Infection risk, including for grade ≥3 infections, tends to be lower with BaAbs targeting GPRC5D (e.g., talquetamab) (25). Although infectious complications are lower with talquetamab compared with BsAbs targeting BCMA × CD3, talquetamab is associated with dysgeusia, skin and nail changes, and xerostomia, which necessitate close monitoring due to their impact on quality-of-life (25).

Finally, hematological adverse events (neutropenia, thrombocytopenia and anemia) are common in patients receiving BsAbs and may be largely managed using supportive strategies and dose delays (52). In patients with grade 3–4 neutropenia, granulocyte colony stimulating factor (G-CSF) is recommended for consideration by the expert panel, consistent with international guidance (13).

### Efficacy outcomes of BsAbs in multiple myeloma

Clinical trial data demonstrates that BsAbs have been associated with high overall response rates (ORRs) as well as progression-free survival (PFS) and OS benefits (20–25). The data from pivotal phase III trials are summarized in Table 2. The comparative efficacy of BsAbs highlights how non-BCMA-targeted BsAbs may have improved response and tolerability compared with BCMA-targeted BsAbs (32). The efficacy profiles of approved agents may therefore be considered in treatment decision-making, as agreed by the expert panel (Box 4).

**Table 2:** Summary of data on BsAbs from pivotal trials.

**Agent**	**Teclistamab**	**Elranatamab**	**Talquetamab**		**Cevostamab**	**Linvoseltamab**
**Trial**	**MajesTEC-1** **(****20**, **21****)**	**MagnetisMM-3** **(****22**, **23****)**	**MonumenTAL-1** **(****25**, **44****)**		**CAMMA-2** **(****26****)**	**LINKER-MM1** **(****27****)**
			**400** μ**g QW**	**800** μ**g Q2W**		
Patients, *N*	165	123	143	154	167	117
Median age (range), years	64 (33–84)	68 (36–89)	62 (46–80)	64 (47–84)	66 (40–90)	70 (37–91)
Median lines of therapy (range)	5 (2–14)	5 (2–22)	6 (2–14)	5 (2–17)	6 (2–18)	5 (2–16)
Triple class refractory patients, %	78	97	74	69	96	82
ORR, %	63	61	74	70	43	71
≥CR, %	46	37	33	40	13	50
MRD-negative, %	81.5^*^	89.7^†^	NA	NA	NA	90.5^‡^
Median DOR, months	24.0	NR (67% at 24 months)	9.5	17.5	10.4	29.4
Median PFS, months	11.4	17.2	7.5	11.2	NA	NR (70% at 12 months)
Median OS, months	22.2	24.6	NA	NA	NA	31.4

^*^MRD negativity rate for patients who were MRD-evaluable were MRD-negative (*n* = 44/54).

^†^MRD negativity rate for patients with ≥CR and who were evaluable for MRD (*n* = 29); equivalent to 60.5% of patients with ≥CR.

^‡^MRD-negativity rate for patients with ≥CR (*n* = 58) and MRD evaluable by clonoSEQ (*n* = 21).

No head-to-head analyses were conducted in the development of this table. The data presented are not intended to imply direct comparative efficacy or safety between the treatments listed.

CR, complete response; DOR, duration of response; MRD, minimal residual disease; NA, not available; NR, not reached; ORR, overall response rate; OS, overall survival; PFS, progression-free survival.

Minimal residual disease (MRD) negativity is recognized as an important prognostic marker in MM; MRD negativity is associated with improved survival (56–58). The expert panel agreed that MRD negativity is an important predictive value for BsAb decision-making in MM.

There is a lack of head-to-head comparisons of BsAbs in patients with MM and hence differences in patient populations and efficacy results across trials should be used with caution to inform treatment decisions, where appropriate (32). Despite international data from clinical trials and emerging real-world evidence (59), the expert panel agreed that there is a need for real-world studies evaluating the use of BsAbs in Saudi Arabia to support regional decision-making.

## Conclusion

This document provides expert recommendations on the optimal use of BsAbs in patients with multiple myeloma in Saudi Arabia. The appropriate use of BsAbs is crucial to maximize benefit and minimize risk to patients globally and in Saudi Arabia. Decision-making should include a detailed evaluation of patient characteristics, MM features, previous therapies, and toxicity profiles of available agents. The sequencing of therapies is a key consideration to ensure patients derive the greatest benefit from BsAbs, particularly as CAR T-cell therapy becomes more accessible in the region. More research, including real-world data on the use of BsAbs, is needed to further refine and guide practice in Saudi Arabia.

## Data Availability

The original contributions presented in the study are included in the article/Supplementary material, further inquiries can be directed to the corresponding author.
