# Supplementary material for: Saudi experts statement on advancing multiple myeloma treatment: the evolving role of bispecific antibodies
Source: Front Med (Lausanne). 2026 Jan 21;12:1730261. doi: 10.3389/fmed.2025.1730261 (PMC12869710; doi:10.3389/fmed.2025.1730261)
Supplement: Supplementary file 1 [file Data_Sheet_1.pdf]

Supplementary Table 1. Virtual survey of defined consensus statements and corresponding levels of agreement on bispecific antibodies in the treatment of multiple myeloma

No.	Statement	Strongly Agree	Tend to Agree	Tend to Disagree	Strongly Disagree	Agreement
**Topic A: Patient selection for bispecific antibodies (BsAbs) in multiple myeloma**						
1	Effective treatment options for triple-class exposed R/R MM represent a significant clinical unmet need					
2	Criteria for selecting patients for bispecific antibodies should include fitness (ECOG performance status), prior therapies, and biomarkers, with special considerations for high-risk cytogenetics and frail patients					
3	The suitability of high-risk patients, such as those with high-risk cytogenetics, early relapse, extramedullary disease, etc., for bispecific antibody therapy should be discussed early, prior to relapse, with the medical team so an agreed pathway can be established					
4	Existing frailty assessment tools (such as ECOG performance status) should be incorporated when developing treatment plans					
5	CAR T-cell treatment is unavailable within Saudi Arabia. When available, patients with rapidly progressing disease may be more suitable for BSAb over CAR T-cell therapy due to the long manufacturing time for anti-BCMA CAR T-cell therapy					
6	Bispecific antibodies should be positioned in triple-class refractory MM. Additional experience is required to inform evidence-based decisions on treatment sequencing for triple-class exposed R/R MM					
**Topic B: Sequencing and dosing of bispecific antibodies (BsAbs) in multiple myeloma**						
1	Optimal dosing and schedule adjustments should be based on patient response and tolerance					
2	Bridging therapy before CAR T-cell therapy should be standardized					
3	Bispecific antibodies (BsAbs) are a preferred option for bridging therapy before CAR T-cell					

	therapy compared to other available methods					
4	Switching the target antigen between bridging therapy and CAR T-cell therapy (e.g., using a GPRC5D-targeting bispecific antibody before BCMA-directed CAR T-cell therapy) may be considered					
5	For patients with R/R MM who have not received CAR T-cell therapy, the optimal treatment option is a BCMA-targeting bispecific antibody (BsAb)					
6	For patients with R/R MM who have previously received BCMA-directed CAR T-cell therapy and subsequently relapsed, a BCMA-targeting bispecific antibody (BsAb) represents an optimal treatment option					
7	For patients with R/R MM who received BCMA-directed CAR T-cell therapy and were bridged with a bispecific antibody (BsAb), switching to a bispecific that targets a different antigen remains a strong treatment option after relapse					
8	In-patient hospitalization is recommended during the initiation phase of bispecific antibody (BsAb) therapy for patients at high risk of cytokine release syndrome (CRS) or neurotoxicity, to ensure prompt recognition and management of adverse events, with outpatient initiation considered only in centers equipped with appropriate monitoring and emergency response capabilities					
9	The up-titration schedule of bispecific antibodies (BsAbs) should follow a gradual escalation approach, particularly during the initial dosing phase (typically the first 2–4 weeks), to minimize the risk of immune-related toxicities such as CRS and ICANS					
10	The transition to maintenance dosing should be based on the patient's response during the up-titration phase, including resolution of any immune-related adverse events (e.g., CRS or ICANS), with careful consideration of any ongoing toxicities					

11	For most bispecific antibodies (BsAbs), the maintenance phase typically involves a reduced dosing frequency following the initial up-titration period, with administration every 2–4 weeks					
12	Patient adherence and quality of life should be prioritized during the maintenance phase, and the dosing schedule should aim to reduce treatment burden without compromising efficacy					
**Topic C: Monitoring of bispecific antibodies (BsAbs) in multiple myeloma**						
1	The incidence and severity of CRS, ICANS, and infections in clinical practice are significant, with long-term safety concerns including cytopenia and immune-related effect					
2	Centers administering bispecific antibodies should establish protocols for monitoring cytokine levels, ferritin, and CRP, to detect early signs of CRS and facilitate timely intervention					
3	CRS is likely to occur soon after initiation (within 1 week) and CRS with late doses is very uncommon					
4	Patients should be aware of the risk of CRS and ICANS prior to initiation of bispecific agents and know who to contact if they experience symptoms					
5	Best practices for grading and management of CRS and ICANS should be established in each center, in line with international guidelines, such as those outlined by the American Society of Hematology (ASH) and the American Society for Transplantation and Cellular Therapy (ASTCT)					
6	During the up-titration phase, patients should be closely monitored for early signs of CRS and ICANS, with assessments performed at frequent intervals (every 4–6 hours), particularly within the first 24–48 hours following each dose escalation					
7	Long-term monitoring during the maintenance phase should include assessments every 1–3 months of disease response (e.g., via bone marrow biopsies, imaging studies, or laboratory markers) and					

	adverse events, such as hematologic toxicity or infections					
8	Hospital ward and on-call medical, ambulatory care, nursing, and pharmacy teams should be familiar with CRS and ICANS treatment and management (including staging and grading)					
9	Biomarkers, such as ferritin, CRP and IL-6, play a role in predicting CRS/ICANS severity					
10	Infections should not be a barrier to treatment. Adequate infection management is vital to avoid treatment interruption. Recurrent infections are common and should be managed prophylactically and cautiously. All patients should receive IVIg and be initiated on antiviral, anti-PCP and antifungal prophylaxis					
11	Bispecific antibody treatment should be started in tertiary centers, and after 1–2 doses the patient can be seen in a secondary hospital					
12	G-CSF is recommended for consideration in patients with Grade 3-4 neutropenia					
**Topic D: Efficacy outcomes of bispecific antibodies (BsAbs) in multiple myeloma**						
1	Given the significant impact of efficacy on treatment outcomes, it is crucial to consider the efficacy profiles of bispecific antibodies when making treatment decisions					
2	The positive outcomes observed in clinical trials, including high overall response rates, prolonged progression-free survival and overall survival underscore the importance of selecting bispecific antibodies with proven efficacy					
3	The predictive value that MRD negativity, evidenced in clinical trials, highlights its importance when selecting bispecific antibodies for treatment decision-making in multiple myeloma					
4	Differences in the patient populations enrolled and efficacy results between various bispecific antibodies have been noted in international randomized controlled trials and real-world evidence that may inform treatment choices					
5	Real-world studies evaluating bispecific antibodies in R/R					

	multiple myeloma need to be prioritized in the patient population in Saudia Arabia					
